# Mitochondria dysfunction, a potential cytoprotection target against ischemia-reperfusion injury in a mouse stroke model

**DOI:** 10.1016/j.neurot.2025.e00549

**Published:** 2025-02-11

**Authors:** Elodie Ong, Paul Clottes, Christelle Leon, Hala Guedouari, Noelle Gallo-Bona, Megane Lo Grasso, Lucas Motter, Radu Bolbos, Michel Ovize, Norbert Nighogossian, Marlene Wiart, Melanie Paillard

**Affiliations:** a Stroke Department, Hospices Civils de Lyon, 69500 Bron, France; b Laboratoire CarMeN - IRIS Team, INSERM, INRA, Université Claude Bernard Lyon-1, Univ-Lyon, 69500 Bron, France; c CERMEP-Imagerie du Vivant, 69500 Bron, France; d CNRS, 69100 Villeurbanne, France

**Keywords:** Cerebral ischemia-reperfusion, PTP, Mitochondrial respiration, Therapeutic

## Abstract

More than 50 ​% of patients undergoing mechanical thrombectomy (MT) for ischemic stroke have a poor functional outcome despite timely and successful angiographic reperfusion, highlighting the need for adjunctive treatments to reperfusion therapy. Mitochondria are key regulators of cell fate, by controlling cell bioenergetics via oxidative phosphorylation (OXPHOS) and cell death through the mitochondrial permeability transition pore (mPTP). Whether these two main mitochondrial functions are altered by reperfusion and could represent a new cytoprotective approach remains to be elucidated in mice. Swiss male mice underwent either permanent or transient middle cerebral artery occlusion (pMCAO or tMCAO), with neuroscore evaluation and multimodal imaging. The area at risk of necrosis was evaluated by per-occlusion dynamic contrast-enhanced ultrasound. Final infarct size was assessed at day 1 by MRI. Cortical mitochondrial isolation was subsequently performed to assess mPTP sensitivity by calcium retention capacity (CRC) and OXPHOS. A cytoprotective treatment targeting mitochondria, ciclosporine A (CsA), was tested in tMCAO, to mimick the clinical situation of patients treated with MT. Reperfusion after 60 ​min of ischemia improves neuroscores but does not significantly reduce infarct size or mitochondrial dysfunction compared to permanent ischemia. CsA treatment at reperfusion mitigates stroke outcome, decreases final infarct size and improves mitochondrial CRC and OXPHOS. Mitochondrial dysfunctions, i.e. reduced mPTP sensitivity and decreased oxygen consumption rates, were observed in pMCAO and tMCAO regardless of the reperfusion status. CsA improved mitochondrial functions when injected at reperfusion. These suggest that both mPTP opening and OXPHOS alterations are thus early but reversible hallmarks of cerebral ischemia/reperfusion.

## Graphical abstract

**Figure:**
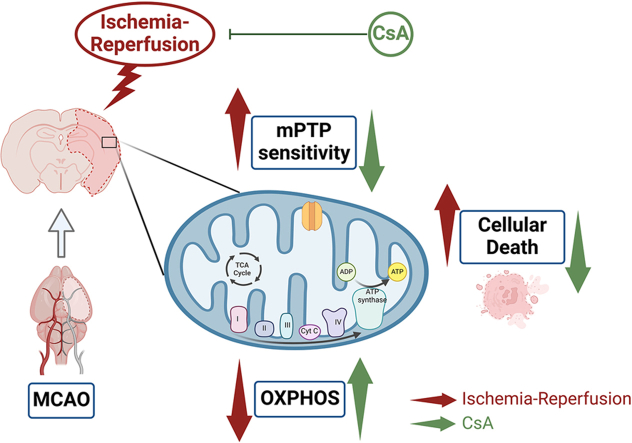

## Introduction

Stroke is the second most common cause of death and a leading cause of disability worldwide: the 30-year projection forecasts an increase of around 30 ​% of its burden in the European Union [1,2]. The target of acute ischemic stroke (AIS) treatment is based on early reperfusion to salvage ischemic penumbra (i.e. the severely hypoperfused, electrically silent, at-risk brain tissue) in order to avoid irreversible brain damage and subsequent neurological disability. Multimodal stroke magnetic resonance imaging (MRI) allows an individual assessment of the initial infarct size, of the site of occlusion and of the hypoperfused region, namely the area at risk of necrosis (AAR). This so-called “ischemic penumbra” is the target of AIS therapies [3]. Intravenous thrombolysis with recombinant tissue plasminogen activator (rt-PA) and mechanical thrombectomy (MT) for selected patients with large vessel occlusion, have demonstrated their efficacy [4]. Nevertheless, more than 50 ​% of patients undergoing MT have a poor functional outcome despite timely and successful angiographic reperfusion [5]. The term “futile reperfusion” is used to describe this condition encompassing the components of the ischemia/reperfusion injury (IRI) including infarct growth and post-treatment complications such as haemorrhagic transformation, malignant edema, and ultimately neurological disability [6]. In fact, sudden reperfusion following thrombectomy promotes ischemic brain damage [7,8]. Although cytoprotective strategies for ischemic stroke have failed to demonstrate efficacy in several randomized clinical trials, the advent of successful reperfusion provides the opportunity to reconsider therapeutic approaches [9]. In the context of MT, IRI remains an important therapeutic issue [9]. To assess candidate cytoprotective therapies against IRI in preclinical studies, the intraluminal transient middle cerebral artery occlusion (tMCAO) murine model is commonly used, since it replicates the prompt recanalization obtained by MT in stroke patients [10,11].

Mitochondria, as the « powerhouse of the cell », contributes to the cell fate notably by maintaining the bioenergetics through oxidative phosphorylation. Accordingly, mitochondria may play a key role in IRI [12]. The mitochondrial permeability transition pore (mPTP), a non-selective pore located in the inner mitochondrial membrane, is a Ca^2+^-activated channel involved in mitochondrial injury and cell death upon stress [13]. During reperfusion after a brain ischemia, a combination of Ca^2+^ overload, oxidative stress and adenosine tri-phosphate lead to mPTP opening and necrotic death [14,15]. Cyclophilin D (CypD) appears to be an essential regulator of the mPTP: a significant reduced infarct size in tMCAO is observed in CypD-deficient mice in which inhibition of mPTP opening has been described [15]. Targeting mPTP opening could be a potential strategy to limit IRI [14]. Cyclosporine A (CsA), a potent in vitro mPTP inhibitor via CypD binding, led to a decreased infarct size in patients with proximal artery occlusion and successful recanalization [16,17].

Whether the two main mitochondrial functions, i.e. oxidative phosphorylation (OXPHOS) and mPTP opening (assessed by calcium retention capacity, CRC, which reflects maximum Ca^2+^ overload that mitochondria can support before mPTP opening [18]), are altered specifically by reperfusion, and could potentially be modulated by a cytoprotective strategy, has not been assessed so far in a mouse model of MCAO to the best of our knowledge.

In this context, the aims of this study were:

(i) To evaluate mitochondrial dysfunctions (using CRC and OXPHOS analysis) in a mouse model of MCAO (transient and permanent) using multimodal imaging (per-occlusion dynamic contrast-enhanced ultrasound (CEUS) assessment of the AAR and MRI at day 1 to evaluate infarct size) and neuroscore evaluation;
(ii) To study CsA as a cytoprotective treatment potentially targeting mitochondria in the tMCAO model combining imaging approach, neuroscores assessment and mitochondrial function analyses (mPTP sensitivity to Ca^2+^ and OXPHOS).

## Materials and methods

### Ethics and animals

All animal experiments were approved by the local review board in Lyon (“Comité d’éthique pour l'Expérimentation Animale Neurosciences Lyon”: CELYNE; CNREEA no. 42; APAFIS agreement no. #24275) and authorized by the French ministry of higher education, research, and innovation. Experiments were performed on 6-week-old male mice (Envigo CD-1®, France), weighting between 25 and 36 ​g on the day of surgery. Mice were acclimated to the animal facility conditions for at least 7 days, housed in a controlled temperature (22 ​± ​2 ​°C) with a 12/12-h light/dark cycle and ad libitum access to pelleted food and water. Humane endpoints were: no reaction to stimuli, prolonged inactivity (>6 ​h), distress signs (stereotypies) and weight loss >15 ​%. These signs were monitored at 1 ​h, 5 ​h and 24 ​h post-surgery. Results are reported according to the ARRIVE guidelines 2.0 [19]. A total of 52 adult male mice were included in this study.

### Study design

**Fig. 1:**
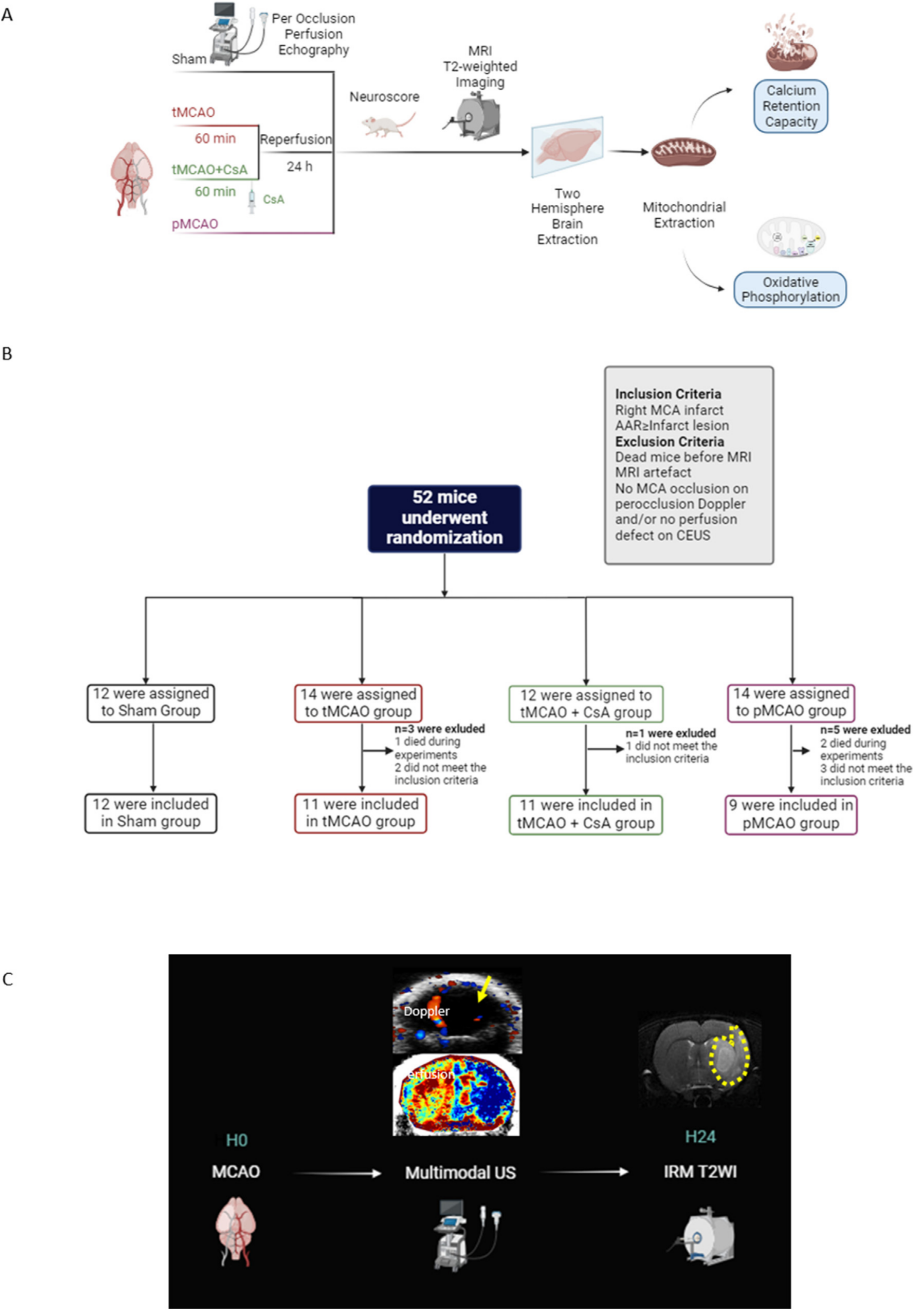
**Description of the study**. A. Study design. Ischemic stroke was induced by middle cerebral artery occlusion (MCAO) using the monofilament model. Mice underwent permanent or transient MCAO (60 ​min) or Sham surgery. Ciclosporin A (CsA) was given in a subgroup of mice with tMCAO. Per-occlusion ultrasound imaging was performed to verify: (i) arterial occlusion using color Doppler mode and (ii) hypoperfusion of the MCA territory using contrast (microbubbles)-enhanced ultrasound. Neuroscores and MRI were obtained 24 ​h post-surgery. Brains were sampled and mitochondria extracted for functional analysis (CRC and OXPHOS). B. Study Flow Chart. A total of 52 animals were randomized. Inclusion/exclusion criteria were defined a priori. MCAO mice that did not have occlusion and/or perfusion defect on per-occlusion ultrasound were excluded. C. Imaging protocol: the yellow arrow shows the right MCA occlusion on Doppler and the perfusion defect appears in blue on CEUS. The yellow dotted line on the MRI delineates the infarct.

Fig. 1A presents the study design. The study adhered to RIGOR guidelines for stroke research [20]. Randomization was performed by block of 3 animals per day (https://www.graphpad.com/quickcalcs/randomize1.cfm). Cofounders such as animal/cage location were not controlled. All experimenters were blinded to the allocation group.

Sample size was calculated a priori using G∗power (Heinrich Heine Düsserldorf Universität, Germany). An alpha level of 0.05 and a power of 0.8 were defined. The primary endpoint was infarct volume measured on T2-weighted MRI at 24 ​h normalized by the area-at-risk of infarction measured on per-occlusion CEUS. We hypothesized a 40 ​% reduction in infarct volume in treatment groups and a variability of 40 ​% according to our previous studies (effect size ​= ​1.21, Mann-Whitney test) [21,22]. The minimal number of animals to be included was determined as 10 mice per group. A 30 ​% exclusion rate (including mortality and exclusion from study or analysis according to a priori-defined criteria as defined below) was anticipated, therefore 52 mice underwent randomization in total.

### Transient and permanent ischemic stroke models

For analgesia, buprenorphine was injected (0.075 ​mg/kg bodyweight; Vetergesic 0.3 ​mg/mL, Alcyon, France) subcutaneously. For induction, mice were anesthetized with 8 ​% sevoflurane. During all procedures, the core temperature of the animals was kept constant at 37 ​°C, using a homothermic blanket heating system. The intraluminal filament model of focal ischemia was used [22,23]. Briefly, under 4 ​% sevoflurane maintenance anesthesia, a size-matched silicon-coated monofilament (Doccol Corporation, USA) was advanced through the right internal carotid artery toward the middle cerebral artery (MCA) junction until resistance was felt (∼8 ​mm). For tMCAO animals, the filament was withdrawn after 60 ​min of occlusion, while it remained in position for pMCAO animals. For Sham animals, the filament was introduced into the MCA but remained not occlusive. CsA (10 ​mg/kg, Sandimmun 50 ​mg/mL diluted in NaCl) was injected in the caudal vein as a bolus (5 ​μl/g body weight) in tMCAO+CsA mice 5 ​min before reperfusion. After suture of the carotid artery access, mice were returned to their respective cage for 24 ​h and were monitored with continuous analgesia (buprenorphine at 0.075 ​mg/kg sc after surgery and on the next morning) until their euthanasia on the next day (cervical dislocation under 2 ​% isoflurane anesthesia).

### Per-occlusion echography imaging

Echography imaging was used per-occlusion to ascertain ischemia (and thus include only animals that were correctly occluded) and to provide a quantitative biomarker of the area at risk of infarction using perfusion imaging. Several acquisitions modes were performed on a Vevo3100 system: B-mode, color doppler and contrast imaging. Per-occlusion dynamic contrast-enhanced ultrasound (CEUS) was performed at 4 slice levels during the infusion of 250 ​μL microbubbles at 50 ​μL/min using the burst-replenishment method (Vevo® 3100 LAZR-X, MX201 probe, Vevo MicroMarker, Visual Sonics) [23].

### Functional status evaluation

Neuroscores were performed 24 ​h after the onset of reperfusion using a 0 to 5 scale score, as previously described [24]. Three points were assessed: parachute reflex, lateral resistance and circling. Mice with no spontaneous motion were assigned a grade of 0. Mice with symmetrical forelimbs extension toward the floor without neurological impairments were assigned a grade of 5. According to the score, we defined three outcome categories: mild (4–5), moderate (2–3) and severe (0–1).

### Magnetic resonance imaging

The MRI protocol was performed on mice using a 7T horizontal-bore Bruker BioSpec Avance I preclinical imaging system (Bruker Biospin MRI GbmH Bruker, Germany). This MRI was equipped with a set of gradients of 440 ​mT/m maximum amplitude and controlled via a Bruker workstation interfaced with ParaVision5.1 software for data acquisition. A Bruker birdcage volume coil (inner diameter ​= ​72 ​mm and outer diameter ​= ​112 ​mm) was used for the signal transmission, and a Bruker single loop surface coil (25 ​mm diameter) was used for signal reception and positioned on the head of the animals to target the brain. Anesthesia was performed with a dedicated system (TEM Sega Lormont, France), induced with a mixture of 3.5 ​% isoflurane gas and air and delivered at a rate of 600 ​mL/min. The animals were placed in supine position in an MRI-compatible mouse cradle provided with a stereotactic holder (Bruker Biospec Animal Handling Systems) and maintained during the entire MRI protocol under anesthesia at 1.5 % isoflurane delivered via a cone mask. The respiratory rate was carefully monitored using a pressure sensor linked to a monitoring device (ECG Trigger Unit HR V2.0, RAPID Biomedical, Rimpar, Germany), and the physiological body temperature was maintained by means of a thermo-regulated water circuit.

The MRI protocol was performed 24 ​h post-MCAO experimental model. To visualize and quantify the post-stroke cerebral vasogenic edema, 2D T2-weighted RARE spin-echo MR images were obtained with the following parameters: field of view (FOV) ​= ​30 ​× ​30 ​mm^2^, matrix size ​= ​256 ​× ​256 pixels, leading to an in-plane spatial resolution of 117 ​× ​117 ​μm^2^, effective echo time (eff-TE) ​= ​75 ​ms, repetition time (TR) ​= ​5000 ​ms, RARE factor ​= ​8, number of averages (NA) ​= ​2, bandwidth ​= ​35 ​kHz. A total of 9 adjacent slices of 1 ​mm thickness were acquired in axial orientation for a 4 ​min scan time, covering the mouse brain from olfactory bulb to cerebellum. Finally, in order to ascertain the middle cerebral artery in pMCAO and recanalization in tMCAO, a 2D time of flight (TOF) with flow compensation angiography images was obtained with the following parameters: field of view (FOV) ​= ​20 ​× ​20 ​mm^2^, matrix size ​= ​192 ​× ​192 pixels, leading to an in-plane spatial resolution of 104 ​× ​104 ​μm^2^, effective echo time (eff-TE) ​= ​3.6 ​ms, repetition time (TR) ​= ​23 ​ms, flip angle: 80 deg, number of averages (NA) ​= ​4, bandwidth ​= ​75 ​kHz. A total of 29 adjacent slices of 0.40 ​mm thickness and 0.25 ​mm interslice distance were acquired in coronal orientation for a 6.24 ​min scan time.

### Inclusion and exclusion criteria

Mice were excluded from the study if they did not have a vascular occlusion on per-occlusion Doppler imaging and/or a perfusion defect as visually assessed on dynamic contrast-enhanced ultrasound. Mice were excluded from analysis if they did not have a complete longitudinal follow-up, if they did not have an infarct in the MCA territory or if they had an infarct lesion superior to the AAR.

### Lesion volume assessment

CEUS analysis: Dynamic images were imported from VevoLab and processed using an in-house Matlab code. Dynamic curves were fitted to the burst-replenishment model to generate perfusion maps. The median value in the contralateral hemisphere was used to normalize perfusion maps on a voxel-by-voxel basis. The AAR was defined automatically as the voxels with hypoperfusion <26 ​% of contralateral hemisphere and its volume is presented as a percentage of the hemisphere. This threshold was defined based on the hypoperfused area that evolved to infarction on T2WI in pMCAO [23].

MRI analysis: For assessment of lesion size, T2WI data were analyzed by a blinded examinator using ImageJ software (National Institute of Health, USA imagej.nih.gox/ij/) by manually contouring the lesion, the ipsilateral and the contralateral hemispheres. Lesion volume was corrected for edema using Gerriets correction and presented as a percentage of the hemisphere, %HLVc [25,26].

### Mitochondrial function study

#### Cortical mitochondrial isolation

The brain was extracted quickly after the sacrifice of the mouse and the two hemispheres were separated. The cerebellum, brain stem and underlying midbrain structures were removed from the forebrain. After mechanical homogenization in an isolation buffer (Mannitol 225 ​mM, Sucrose 75 ​mM, Hepes 20 ​mM, EGTA 0.1 ​mM, BSA 0.5 ​%, pH 7.4), a first centrifugation was performed at 500 ​g for 5 ​min at 4 ​°C to cast out the unwanted membranes and organelles, and then a second centrifugation at 9000 ​*g* for 10 ​min at 4 ​°C to pellet mitochondria. Mitochondria were finally suspended in a storage buffer (Mannitol 225 ​mM, Sucrose 75 ​mM, Hepes 10 ​mM, pH 7.4) and kept on ice for a maximum of 4 ​h. Final protein content was assessed with the Lowry method before subsequent analyses.

#### Calcium retention capacity

For CRC, we used two Hitachi Research Fluorescence Spectrophotometer (F-2500 and F-7000). 500 ​μg of isolated cortical mitochondria from each hemisphere were suspended in 2 ​mL of buffer at 25 ​°C (150 ​mM Saccharose, 50 ​mM KCL, 2 ​mM KH_2_PO_4_, 5 ​mM Succinate in 20 ​mM Tris/HCl, pH 7.4) with 0.5 ​μM Calcium Green in the spectrophotometer cuvette to follow the extramitochondrial Ca^2+^ level. After a 2-min phase of stabilization, we performed repetitive pulses (every minute) of 10 ​μL CaCl_2_ at 500 ​μM until the burst of Ca^2+^ by mitochondria, corresponding to mPTP opening. The results were expressed in nmol of Ca^2+^ per mg of mitochondria.

#### Oxidative phosphorylation (OXPHOS)

To determine the oxygen consumption rates (OCR) through the different complexes, a successive substrate-inhibitor titration protocol was performed on a high-resolution oxygraph (Oxygraph-2k; Oroboros, Innsbruck, Austria) at 25 ​°C. 500 ​μg of isolated mitochondria from each hemisphere were added in 2 ​mL of respiration buffer (100 ​mM KCL, 50 ​mM Mops, 1 ​mM EGTA, 5 ​mM Kpi, pH 7,4 with 1 ​mg/mL of bovine serum albumine without fatty acid) in each cuve. Addition of glutamate/pyruvate/malate (final concentration 3 ​mM) followed by ADP (2 ​mM) led to the measurement of OCR after complex I stimulation. Successive additions of rotenone (complex I inhibitor, 0.5 ​μM), succinate (complex II substrate, 10 ​mM), TTFA (complex II inhibitor, 40 ​μM), TMPD/ascorbate (complex IV substrate, 0.25/2.5 ​mM) and azide (complex IV inhibitor, 1.5 ​mM) allowed determination of sensitive rates of oxidative phosphorylation after stimulation of complexes II and IV. Data were analyzed with the Oroboros DatLab4 software and expressed as nanomoles of oxygen per minute per milligram of proteins.

### Statistical analysis

GraphPad Prism 4 (Graph Pad Software Inc, La Jolla, CA) was used for statistical analysis. Due to the effective per group, data were analyzed by Kruskal-Wallis and Mann-Whitney tests. Data are presented as Median [interquartile range 25 ​%; 75 ​%]. A p value ​< ​0.05 was considered significant. Treatment-effect modification on neuroscores was evaluated in subgroups of mice (mild, moderate, and severe): the chi-square test of proportions (with a two-sided alpha level of 5 ​%) was used to determine the between-group differences.

## Results

Study flow chart is displayed on Fig. 1B and imaging protocol on Fig. 1C. Fifty-two mice were randomized (n ​= ​12 Sham, n ​= ​14 tMCAO, n ​= ​12 tMCAO ​+ ​CsA, n ​= ​14 pMCAO). Ten mice were excluded from analysis: 6 mice did not meet the inclusion criteria and 3 did not have a complete follow-up because they died before MRI at 24 ​h (2 in pMCAO and 1 in tMCAO groups).

### Reperfusion after 60 ​min of ischemia improves neuroscores but does not significantly reduce infarct size compared to permanent ischemia

**Fig. 2:**
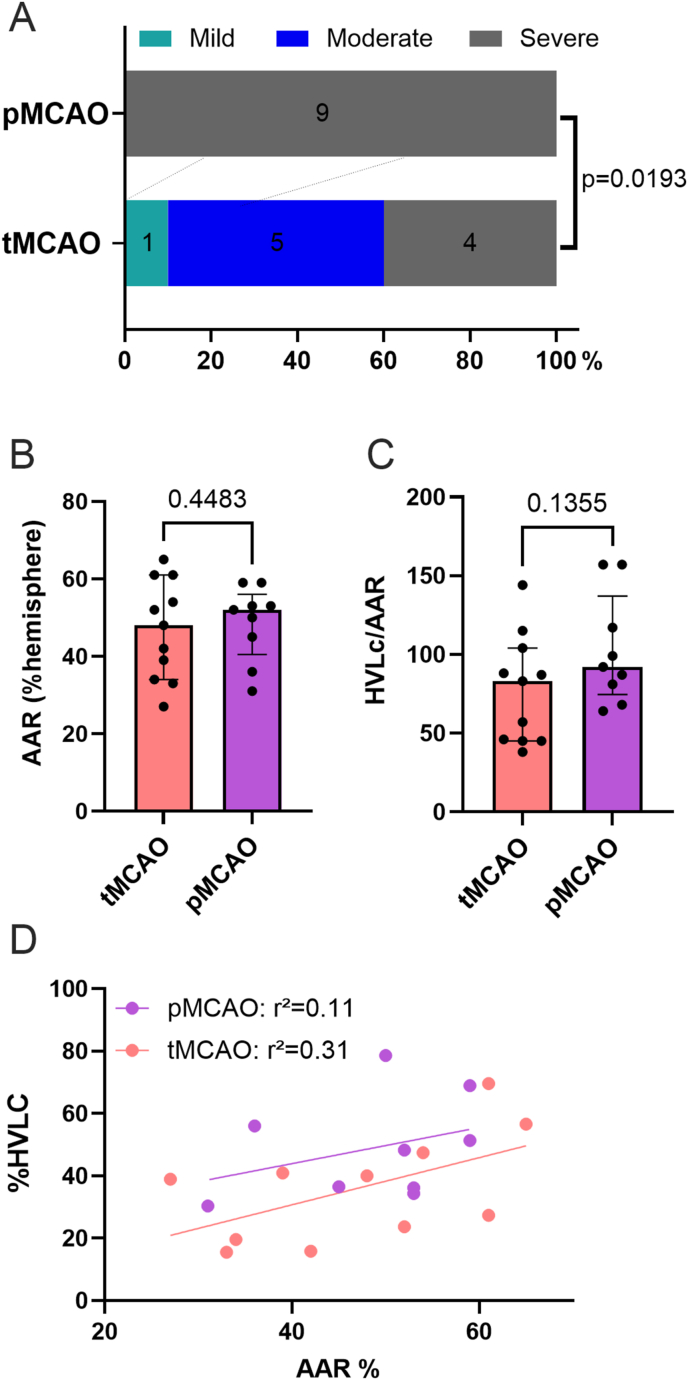
**Comparison between transient middle cerebral artery (tMCAO) and permanent MCAO (pMCAO) mice**. A. Neuroscores in the two groups, tMCAO and pMCAO. Three outcome categories were defined based on the Bederson score (0–5): mild (4–5), moderate (2–3) and severe (0–1). One mouse from the tMCAO group and 2 mice from the Sham group did not get a neuroscore because of neurobehavior experimenter unexpected absence. Neuroscores were significantly improved in tMCAO compared to pMCAO. Chi-square test. B. Quantification of the area at risk (AAR) in tMCAO and pMCAO groups. The AAR was defined as the hypoperfused region on per-occlusion contrast-enhanced ultrasound. As expected, AAR were not statistically different between tMCAO and pMCAO. Mann-Whitney test, Median [interquartile range 25 ​%; 75 ​%]. C. Quantification of the infarct size calculated as the ratio of lesion volume measured on T2-weitghed MRI over AAR (%HLVc/%AAR) in tMCAO and pMCAO groups. Infarct sizes were not different between groups. Mann-Whitney test, Median [interquartile range 25 ​%; 75 ​%]. D. Lesion volume presented as percentage of the hemisphere (%HVLc) relative to the AAR, showing that for a given AAR, infarct size was lower for tMCAO mice, although this was not significant (p ​= ​0.11).

After reperfusion, tMCAO mice displayed significantly improved neuroscores in comparison with pMCAO mice (p ​= ​0.0193) (Fig. 2A). Importantly, the AAR was similar in tMCAO and pMCAO groups (48 ​% [34 ​%; 61 ​%] vs 52 ​% [41 ​%; 56 ​%] p ​= ​0.45) (Fig. 2B). The primary endpoint (%HLVc/AAR) revealed no significant difference between tMCAO and pMCAO groups (83 ​% [45; 104]) vs 92 ​% [75; 137] p ​= ​0.14) (Fig. 2C). For a given AAR, the lesion size was not statistically different between tMCAO and pMCAO model (Fig. 2D) (p ​= ​0.1107).

### Sixty minutes of ischemia with 24h of reperfusion adversely affects mitochondrial CRC and OXPHOS

**Fig. 3:**
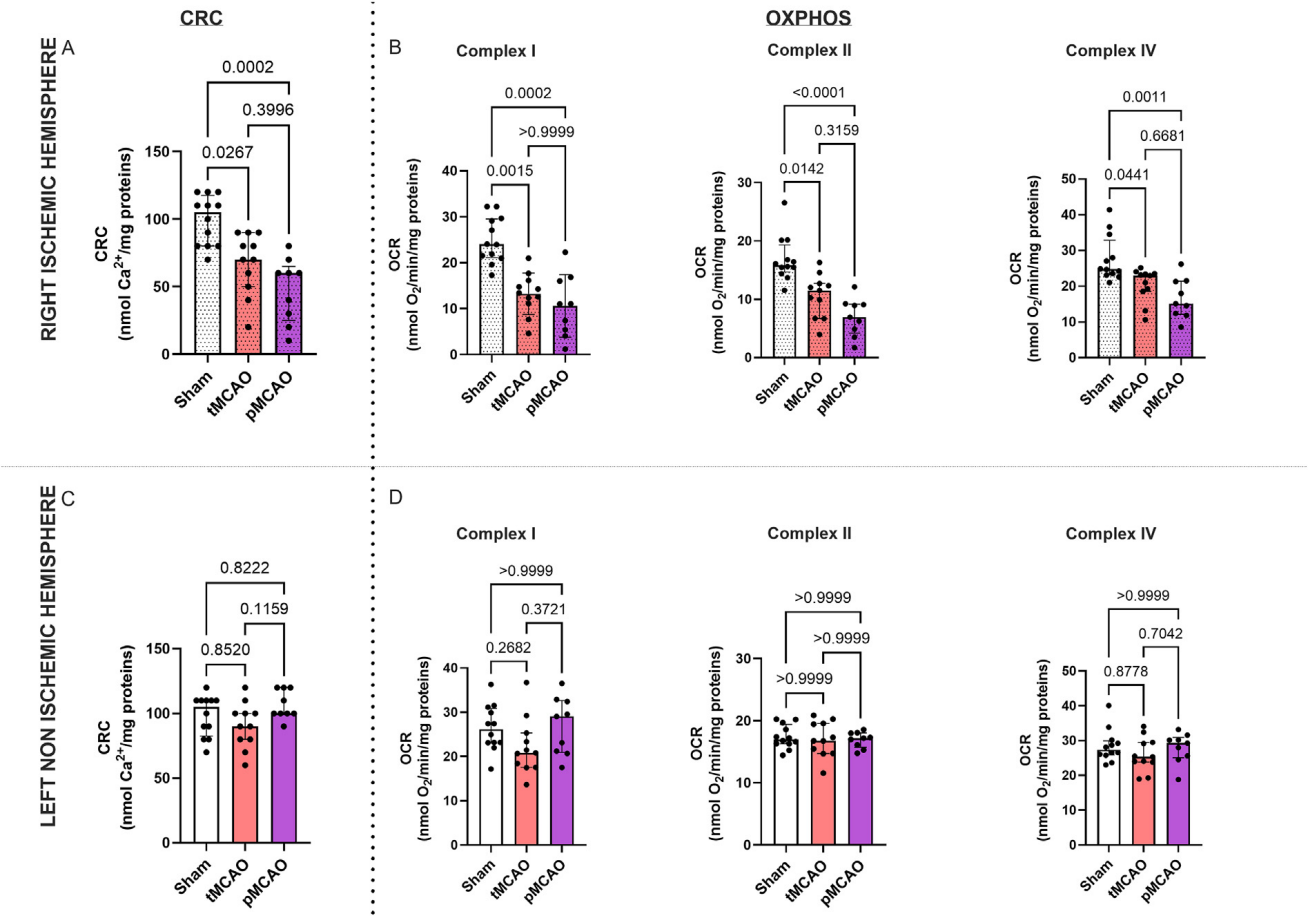
**Comparison of mitochondrial functions between sham, transient middle cerebral artery (tMCAO) and permanent MCAO (pMCAO) mice**. Upper figures: right ischemic hemisphere. A. Calcium Retention Capacity (CRC), B. OXPHOS after stimulation of complex I, II or IV. Bottom figures: left non-ischemic hemisphere. C. CRC, D. OXPHOS after stimulation of complex I, II or IV. Data are expressed as Median [interquartile range 25 ​%; 75 ​%]; Kruskal-Wallis test. As expected, no statistical difference was found between groups in the non-ischemic hemisphere. In contrast, CRC and OXPHOS were decreased in the ischemic hemisphere in MCAO groups vs Sham, with no statistically significant difference between permanent and transient MCAO.

We next investigated the impact of reperfusion on mPTP opening and oxidative phosphorylation. Mitochondria were extracted in both groups from the left and right hemispheres. On mitochondria isolated from the right ischemic hemisphere, we observed a significantly decreased CRC in pMCAO and tMCAO compared to Sham mitochondria (pMCAO: 60 [25; 65] and tMCAO: 70 [50; 90] versus Sham: 105 [80; 118] nmol Ca^2+^/mg protein) (Fig. 3A): no further difference was measured in CRC between pMCAO and tMCAO ischemic mitochondria. In the left non-ischemic hemisphere, CRC reached the same level between groups, averaging 100 ​nmol Ca^2+^/mg protein (Fig. 3C). While no difference was observed in CRC between the right and left hemisphere in the Sham group, CRC was lower in mitochondria isolated from the right ischemic hemisphere compared to their respective left non-ischemic hemisphere in the tMCAO group (p ​< ​0.0059) and in the pMCAO group (p ​< ​0.0039).

Concerning the OXPHOS analysis, we quantified the oxygen consumption rates induced by the stimulation of the different electron transport chain complexes. We found a significant difference between Sham and pMCAO groups respectively for complex I (24 [21.1; 29.5] vs 11 [4.8; 16.4] nmol O_2_/min/mg, p ​= ​0.0002), II (15.9 [14.7; 19.3] vs 6.93 [4.2; 9.2] nmol O_2_/min/mg, p ​< ​0.0001) and IV (24.8 [23.2; 32.9] vs 15.1 [12.1; 21.4] nmol O_2_/min/mg, p ​= ​0.0011) respiratory rates in mitochondria from the right ischemic hemisphere (Fig. 2B). Oxygen consumption rates were significantly decreased in the pMCAO ischemic mitochondria compared to the Sham ones, whatever the substrates used. In the tMCAO group, respiratory rates in the right ischemic mitochondria hemisphere were significantly decreased compared to the Sham group after addition of substrates of either complex I (tMCAO: 13.4 [11.1; 16.2] vs Sham: 24 [21.1; 29.5] nmol O_2_/min/mg, p ​= ​0.0015), II (tMCAO: 15.9 [14.7; 19.3] nmol O_2_/min/mg, vs Sham: 11.5 [6.7; 12.7] nmol O_2_/min/mg, p ​= ​0.0142) or IV (tMCAO: 24.8 [23.2; 32.9] vs Sham: 22.99 [18.6; 23.5] nmol O_2_/min/mg, p ​= ​0.0441). However, no significant difference in oxygen consumption rates was observed between tMCAO and pMCAO ischemic mitochondria. Moreover, no differences were observed between groups regarding respiratory rates for each complex in mitochondria isolated from the non-ischemic left hemisphere.

In short, our data showed that similar anomalies, i.e. increase of mPTP sensitivity to Ca^2+^ and alteration of OXPHOS in the right ischemic hemisphere, were induced in both pMCAO and tMCAO models.

### CsA treatment at reperfusion improves stroke outcome and decreases final infarct size

**Fig. 4:**
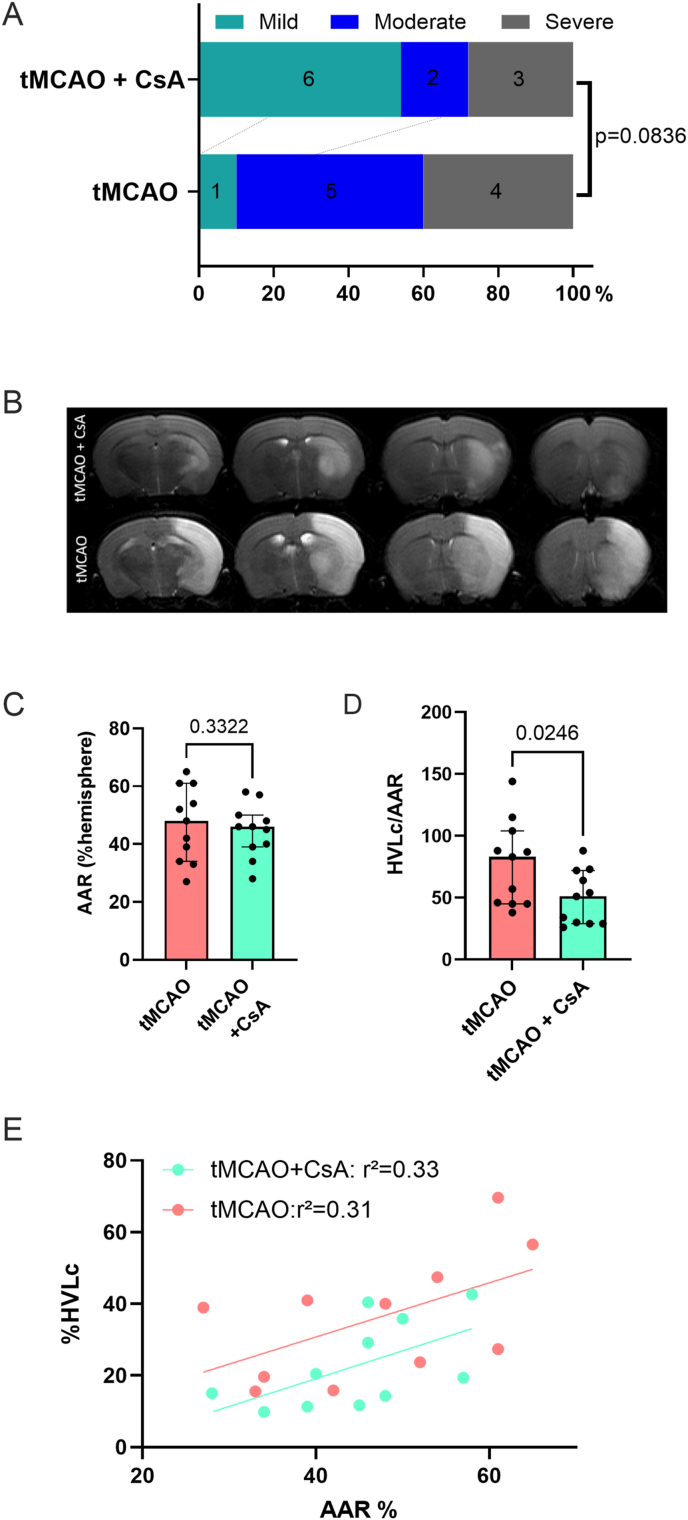
**Effect of Cyclosporine (CsA) treatment in mice with transient middle cerebral artery (tMCAO)**. A. Neuroscores in tMCAO and tMCAO+CsA groups. Neurofunctional outcome was improved in CsA-treated mice although statistical significance was not reached. B. Representative infarct lesions in tMCAO and tMCAO ​+ ​CsA groups (4 central slices on T2-weitghed MRI). C. Quantification of the area et risk (AAR) in tMCAO and tMCAO ​+ ​CsA groups. As expected, AAR were not statistically different between tMCAO and pMCAO. Mann-Whitney test, Median [interquartile range 25 ​%; 75 ​%]. D. Quantification of the infarct size calculated as the ratio of lesion volume over AAR (%HVLc/%AAR) in tMCAO and tMCAO ​+ ​CsA groups. The primary endpoint was statistically lower in CsA-treated animals. Mann-Whitney test, Median [interquartile range 25 ​%; 75 ​%]. E. Lesion volume presented as percentage of the hemisphere (%HVLc) relative to the area at risk (%AAR). For a given AAR, infarct size was significantly lower for CsA-treated mice (p ​= ​0.0494).

To evaluate the potential reversibility of mitochondrial dysfunction induced by tMCAO, we chose a well-known cytoprotective drug that modulates in vitro mitochondrial function, CsA. tMCAO mice received a single bolus of 10 ​mg/kg CsA 5 ​min before reperfusion. Neuroscores tended to improve in the tMCAO+CsA vs tMCAO mice (tMCAO+CsA: 4 [1; 4] vs tMCAO: 2 [1; 3], p ​= ​0.0836) (Fig. 4A). Fig. 4B presents representative infarct lesions from tMCAO+CsA and tMCAO groups. No differences were observed in AAR between groups (tMCAO: 48 ​% [34 ​%; 61 ​%] vs tMCAO+CsA: 46 [39 ​%; 50 ​%]) (Fig. 4C). tMCAO+CsA mice displayed a significantly decreased infarct size (tMCAO+CsA: 51 ​% [29 ​%; 72 ​%]) vs tMCAO: 83 ​% [45 ​%; 104 ​%] %HLVc/AAR, p ​= ​0.02) (Fig. 4D). For the same AAR, the lesion size in the tMCAO ​+ ​CsA group lies below the regression line of the tMCAO mice, suggesting significantly smaller lesion size compared to tMCAO mice (Fig. 4E, p ​= ​0.0494).

### CsA treatment at reperfusion improves mitochondrial CRC and OXPHOS

**Fig. 5:**
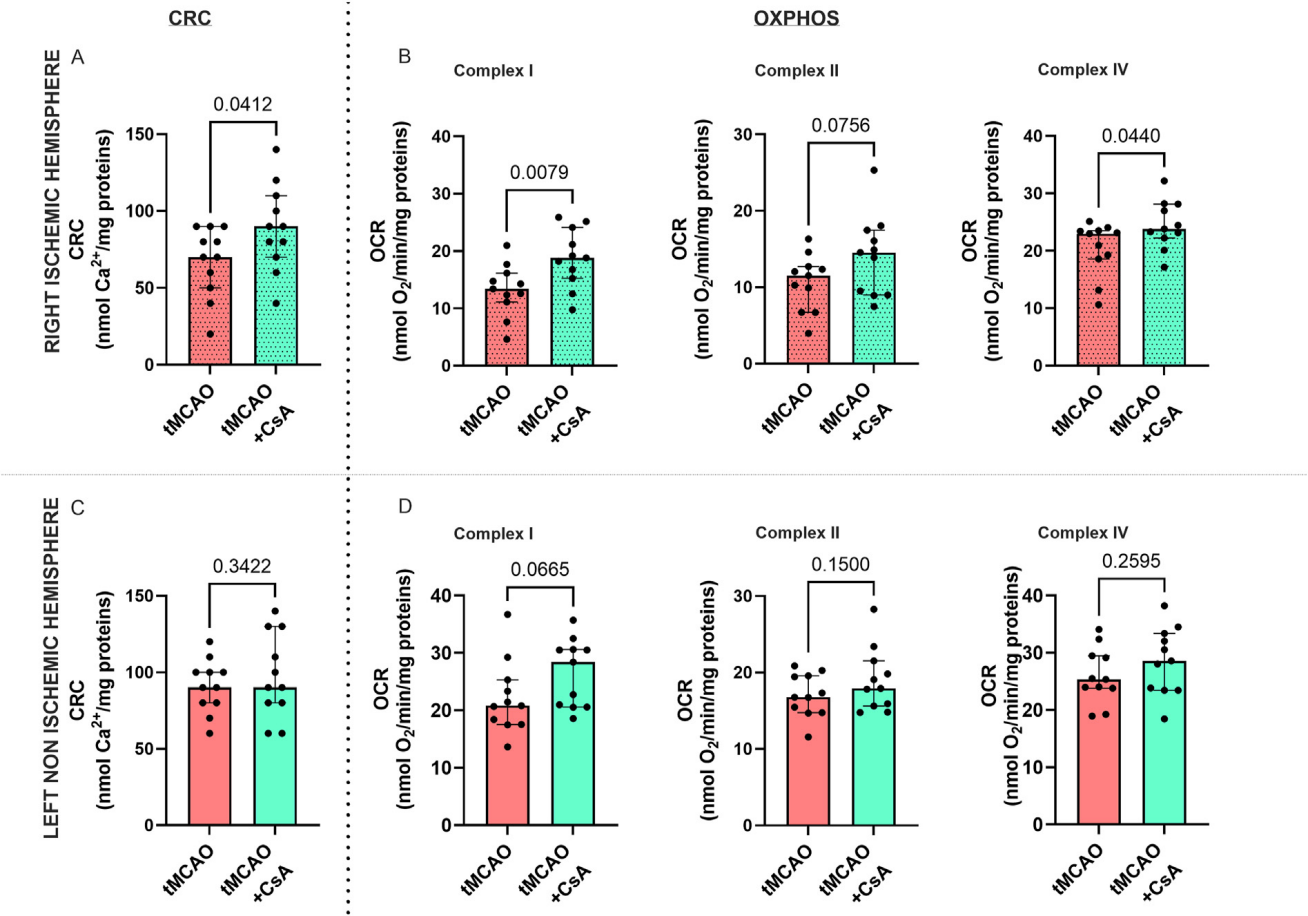
**Effect of CsA treatment on mitochondrial function in mice with transient middle cerebral artery (tMCAO)**. Upper figures: right ischemic hemisphere. A. Calcium Retention Capacity (CRC), B. OXPHOS after stimulation of complex of I, II or IV. Bottom figures: left non-ischemic hemisphere. C. CRC, D. OXPHOS after stimulation of complex I, II or IV. Data are expressed as Median [interquartile range 25 ​%; 75 ​%]; Mann-Whitney test. As expected, no statistical difference was found between groups in the non-ischemic hemisphere. In contrast, CRC and OXPHOS were improved in the ischemic hemisphere of CsA-treated mice.

**Fig. 6:**
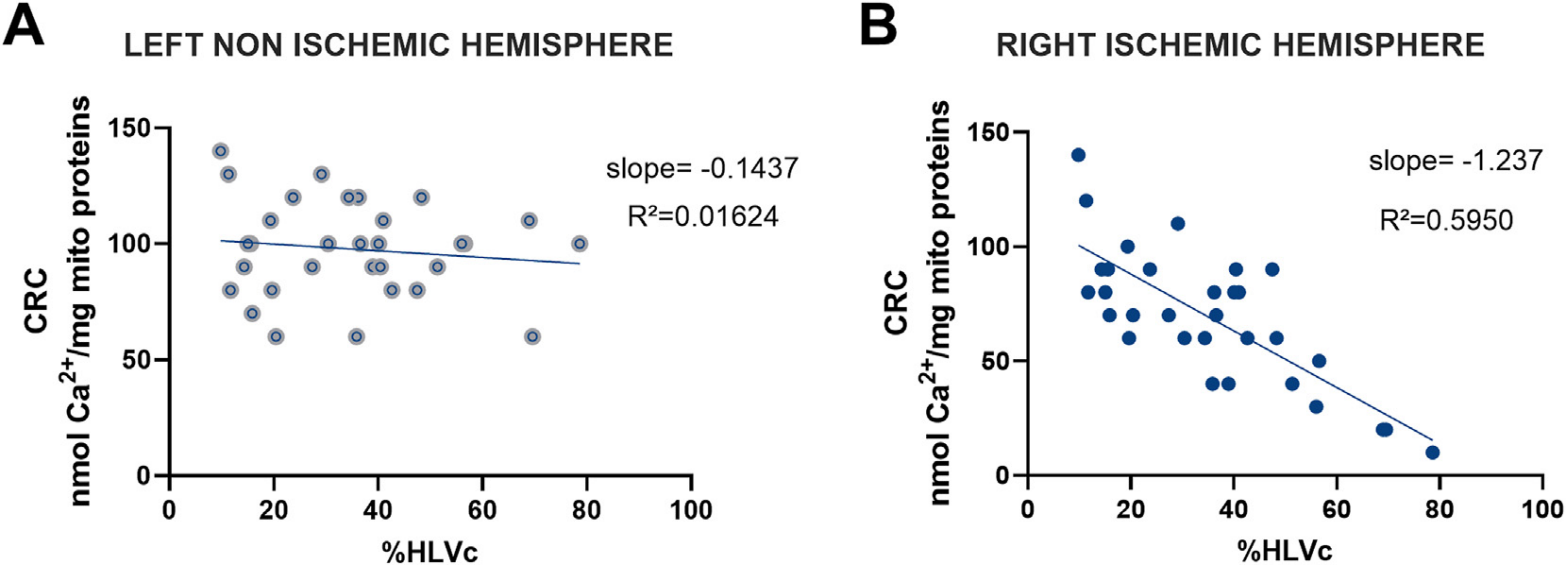
**Correlation between CRC and %HVLc in the left non-ischemic hemisphere (A) and right ischemic hemisphere (B)**. There was no relationship between CRC and infarct size in the non-ischemic hemisphere. In contrast, CRC was negatively correlated to infarct size in the ischemic hemisphere.

A significant increase of CRC in the tMCAO+CsA group vs tMCAO mice was observed for the ischemic mitochondria (tMCAO ​+ ​CsA: 90 [70; 110] vs tMCAO: 70 [50; 90] nmol Ca^2+^/mg, p ​= ​0.0412) (Fig. 5A). Concerning OXPHOS, we measured in tMCAO+CsA ischemic mitochondria when compared to tMCAO group a significant improvement of respiration with complex I substrate (18.8 [15.2; 24.1] vs 13.4 [11.2; 16.2] nmol O_2_/min/mg, p ​= ​0.0079) (Fig. 5B). This was also true for complex IV substrate, albeit to a lesser extent (23.8 [22.2; 28.2] vs 23.0 [18.6; 23.5] nmol O_2_/min/mg, p ​= ​0.044) (Fig. 5B). There was no difference of either CRC or OCR in the left hemisphere mitochondria regardless of the group (Fig. 5C–D), except a trend toward an improved complex I-linked respiration by CsA (p ​= ​0.0665, Fig. 5D). We finally investigated if the lesion size could correlate with the mPTP sensitivity. In the right ischemic hemisphere, there was a significantly negative correlation between lesion size and CRC, i.e. CRC in ischemic mitochondria decreases as lesion size increases (Fig. 6B). On the other hand, no correlation was observed between CRC and lesion size in the left hemisphere (Fig. 6A).

## Discussion

In the present study, we investigated the impact of cerebral ischemia-reperfusion on the two main mitochondrial functions involved in both cell fate and bioenergetics (mPTP sensitivity to Ca^2+^ overload and OXPHOS analysis) in a mouse model of thrombectomy. Mitochondrial dysfunctions have been well addressed in ischemia-reperfusion models in heart and kidney [27,28], but has been less explored in ischemic stroke, notably in mouse models. Our study describes mitochondrial alterations observed in a mouse model of focal cerebral ischemia. The characterization of the two models, pMCAO versus tMCAO with MT, using a novel imaging approach combining ultrasound and MRI methods, is also valuable for the stroke research community with the ambition of improving the quality of preclinical experiments. The study of mitochondrial functions in parallel sheds new light on the physiopathology of IRI damage.

Here, we observed that in comparison to Sham-operated mice, pMCAO mice had an increased mPTP sensitivity to Ca^2+^ and an OXPHOS dysfunction (after stimulation of either complex I, II or IV), as expected. Surprisingly, reperfusion did not significantly improve these markers of mitochondrial dysfunction. In line with these results, final infarct sizes of tMCAO mice did not statistically differ from that of pMCAO. In contrast, neuroscores were statistically improved in tMCAO vs pMCAO groups. Altogether, these results suggest that despite a neurofunctional benefit, reperfusion could at the same time promote mitochondrial injuries. This is concordant with previous studies that used different experimental protocols or mitochondrial endpoints [[29], [30], [31]]. We thus hypothesized that we could prevent post-reperfusion mitochondrial injuries and consequently improve stroke outcome.

Several therapeutic strategies at reperfusion, called post-conditioning, have been evaluated in ischemic stroke. Post-conditioning with interruptions of reperfusion in a rat model reduces infarct size [32]. Local post-conditioning directly in a brain artery is compromised in clinical practice because it is unsafe and operationally difficult. However, pharmacological post-conditioning is easier to use in clinical routine. CsA is known to inhibit mPTP opening [17,22]. A clinical study assessing CsA in stroke showed a significant decrease in cerebral infarct size in a subgroup of patients who received thrombolysis with successful recanalization, as this trial was performed before the onset of MT [16]. This promising study motivated us to focus on mitoprotection by CsA to elucidate the mechanism of action before going back from bench to bedside. In the present study, CsA improved mPTP sensitivity and OXPHOS, decreased infarct size and further improved neuroscores at day 1. Recently, CsA was shown to reduce cerebral inflammation in a non-human primate model of stroke with MT [33]. Altogether, these data suggest that preventing mitochondrial dysfunction is a relevant therapeutic target in cerebral ischemia-reperfusion and supports the launching of a new clinical trial assessing CsA protective effect in MT-treated ischemic stroke patients.

To address methodological flaws commonly identified in preclinical stroke studies, we implemented a robust methodology that included a priori sample size calculation, randomization, blinding and longitudinal imaging. Multimodal echography (Doppler and perfusion) was performed during the occlusion: (i) to select animal with effective MCAO and (ii) to account for individual variability in arterial collaterality by normalizing the final infarct size to the initial hypoperfused area, each animal being its own control. A previous study showed that MRI may be used to replace 2,3,5-triphenyltetrazolium chloride (TTC) post-mortem staining for assessing infarct size at day 1 [21]. By using MRI to assess final infarct size, we were able to study mitochondrial function in animals for which infarct size was controlled. This improves the results’ robustness and has the advantage to reduce the number of animals. Interestingly, thanks to this approach, we were able to demonstrate a significant correlation between the PTP sensitivity in the ischemic hemisphere and final infarct size. We complemented our approach by a neurofunctional evaluation using neuroscores at day 1. This methodology is original and may become the gold standard to study cytoprotective drugs targeting mitochondria.

Nevertheless, our study presents some limitations. Surgery was performed on male young mice, without comorbidities. Since mitochondria were isolated from the whole hemisphere, we cannot address if they belong to grey matter or white matter, as these tissues have a different tolerance according to the severity of ischemia [34]. While we did not perform a dose-response study of CsA effect, we chose a validated dosage of CsA in post-conditioning as previously reported [27]. The duration of 60 ​min of ischemia and 24 ​h of reperfusion was chosen to study acute secondary ischemic injury only [35]. It would be important in a future work to evaluate whether CsA benefits are sustained on the long term. Finally, we cannot assert if CsA action was acting directly on the mPTP or via its known effect on calcineurin [36,37]. Some previous studies also mentioned that CypD could regulate OXPHOS activity notably by modifying respirasome assembly [38].

In conclusion, mitochondrial dysfunctions represented by early mPTP opening and OXPHOS alterations were observed in pMCAO and tMCAO mice regardless of the reperfusion status. CsA improved mitochondrial functions when injected at reperfusion. Therefore, these mitochondrial dysfunctions are partially reversible using a cytoprotective therapeutic approach targeting mitochondria. As time windows for the treatment of AIS have widened, drug and mechanical reperfusion therapies could be combined with cerebral cytoprotective techniques in the future, a promising approach to prevent ischemic damage associated with ischemia-reperfusion injury.

## Author Contributions

Conceptualization: MW, MP.

Data curation: LM, EO, PC, MW, MP.

Formal analysis: LM, EO, PC.

Funding acquisition: MO, NN, MW, MP.

Investigation: EO, PC, HG, NGB, CL, MLG, RB.

Methodology: MO, NN, MW, MP.

Project administration: MW, MP.

Resources: HG, NGB, CL, MLG, RB.

Software: LM.

Supervision: MW, MP.

Validation: MO, NN, MW, MP.

Visualization: LM, EO, PC.

Writing – original draft: EO, MW, MP.

Writing – review & editing: All authors.

## Data availibility

The processed data required to reproduce these findings and perform the statistical analyses are available for download at the figshare repository: https://figshare.com/s/59226fa3098ae31726da.

## Funding

This project was funded by the OPeRa IHU research program (ANR-10-IBHU-0004) to MO, the Leducq Transatlantic Network of Excellence “Targeting mitochondria to treat heart disease” (MitoCardia, 16CVD04) to MP and MO, a grant from the Agence Nationale de la Recherche (ANR- 20-CE14-0013-01) to MP, and a grant from the Agence Nationale de la Recherche (ANR-18-CE19-0003) to MW.

## Declaration of competing interest

The authors declare the following financial interests/personal relationships which may be considered as potential competing interests: Melanie Paillard reports financial support was provided by French National Research Agency. If there are other authors, they declare that they have no known competing financial interests or personal relationships that could have appeared to influence the work reported in this paper.
